# Pancolitis with Ischemic Injury as a Complication of Immunosuppressive Treatment in a Patient with Autoimmune Hepatitis: A Case Report

**DOI:** 10.1155/2012/698404

**Published:** 2012-12-31

**Authors:** A. Dalbeni, E. Capoferro, L. Bernardoni, P. Capelli, A. Caliò, A. Gabbrielli, F. Capra

**Affiliations:** ^1^Section of Internal Medicine, Department of Biomedical and Surgical Science, University of Verona, 10-37134 Verona, Italy; ^2^Section of Gastroenterology, Department of Biomedical and Surgical Science, University of Verona, 10-37134 Verona, Italy; ^3^Section of Endoscopy, Department of Biomedical and Surgical Science, University of Verona, 10-37134 Verona, Italy; ^4^Section of Pathology, Department of Biomedical and Surgical Science, University of Verona, 10-37134 Verona, Italy

## Abstract

Ischemic colitis is a serious drug-induced adverse event. There are only few cases of immunosuppression-associated ischemic colitis described in the literature, but none with a pancolitis-like manifestation. We report the case of a 72-year-old female patient who developed a pancolitis with ischemic injury on immunosuppressive treatment with steroids and azathioprine for autoimmune hepatitis. The patient presented with massive rectal bleeding. Colonoscopy confirmed the diagnosis of pancolitis. The results of histological examination indicated drug-induced ischemic colitis involving the entire colon. This is the first case of ischemic pancolitis mimicking an inflammatory bowel disease (IBD) in a patient with immunosuppressive therapy.

## 1. Background

Ischemic colitis is a serious drug-induced adverse event. However, the exclusion of other causes of ischemic colitis, such as hypotension, vascular surgery, and hypercoagulopathy, is crucial before attributing ischemic colitis to drugs [1].

Ischemic colitis is a disease caused by ischemia of the intestinal vessels, which occurs in patients older than 50 years of age and with many vascular comorbidities. It presents with sudden onset of abdominal pain (80% cases), diarrhoea, and hematochezia, but also abdominal distension, leukocytosis, shock, and sepsis.

Drugs can cause ischemic colitis by producing vasoconstriction (cocaine, dopamine), decreasing splanchnic flow via systemic hypotension (diuretics, Ace inhibitors), vasculitis (gold compounds), or promotion of thrombosis from hormonal effects (estrogen) [1].

Diagnosis is confirmed by colonoscopy, and the major findings are mucosal bleeding, edema, and longitudinal ulcers [2]. The thickening of the bowel wall detected by ultrasonography and computed tomography are useful as another supportive tests in the diagnosis of ischemic colitis. We describe a case of an elderly woman who developed ischemic colitis during immunosuppressive therapy with steroids and azathioprine. There are only few cases of immunosuppression-associated ischemic colitis described in the literature, but none with a pancolitis-like manifestation.

## 2. Case Report

A 72-year-old woman with a recent diagnosis of type 1 autoimmune hepatitis was admitted to our hospital for a three-day history of lower abdominal pain associated with rectal bleeding.

In December 2011, the patient was admitted for the first time to our unit with marked fatigue and blood results suggestive of acute hepatitis (ALT = 1152 IU/L, AST 755 IU/L, total bilirubin 2.95 mg/dL, and directed bilirubin 2.13 mg/dL). Viral markers (HAV, HBV, HCV, CMV, and EBV) were negative, while ANA and ASMA were positive (1 : 320 with homogeneus pattern and 1 : 80, resp.). AMA and anti-LKM were negative.

A liver biopsy has been resulted compatible with a diagnosis of autoimmune hepatitis.

Prednisone was started at dose of 1 mg/Kg/die and azathioprine at a dose of 100 mg/die. After 10 days, the blood results were within the normal range, and the patient was symptoms free.

The steroid dose was gradually decreased. The patient felt well until February 2012 when she was readmitted for abdominal pain and rectal bleeding. At this time, she was on 0.5 mg/Kg/die of Prednisone and 100 mg/die of azathioprine. On admission to our unit, blood pressure was 150/90 mm Hg, pulse was 90 bpm, and temperature was 36.4°C. Her BMI was 25. No abuse of drugs was reported. Physical examination was normal. Laboratory values on admission were haemoglobin 12.1 g/dL, haematocrit 38%, MCV 94 fl, white cell count 10.130/cc, platelet count 202 000/cc, creatinine 0.69 mg/dL, total bilirubin 1.02 mg/dL, GGT 24 IU/L, AST 11 IU/L, ALT 36 IU/L, and LDH 458 IU/L; protein C reactive was 4 mg/dL, while procalcitonin and other inflammatory markers ware negative. Also blood cultures and stool cultures were negative. The coagulation tests like protein C, protein S, and antithrombin III, factor V Leiden mutation, and prothrombin 20210G/A mutation were all negative.

**Figure 1:**
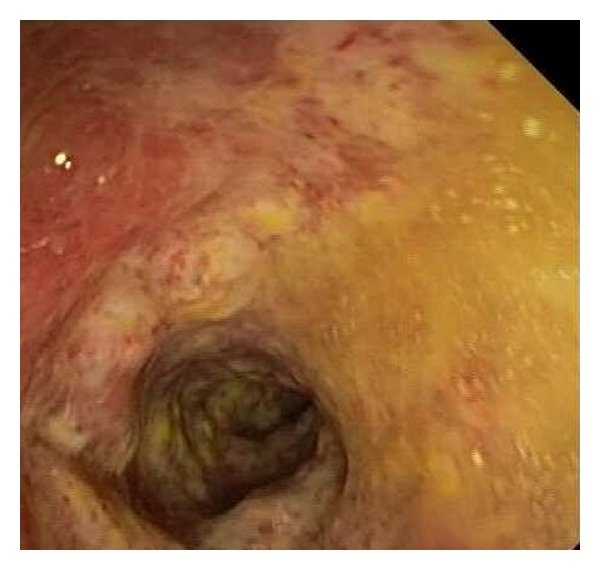
Colonscopy revealed severe active pancolitis.

On the day after admission, the patient underwent colonoscopy (see Figure 1) which revealed severe active pancolitis (from rectal mucosa to all the colon), severe mucosal edema and erythema, multiple erosions, and rare ulcerations. An ultrasound showed thickening of the bowel wall.

**Figure 2:**
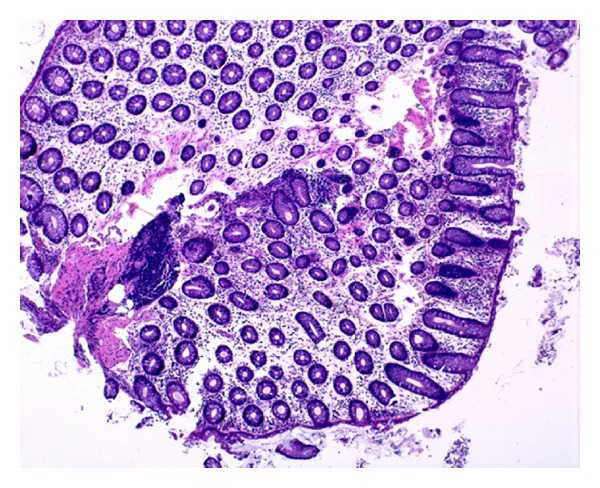
The histological examination shows a modest and diffuse ischemic injury involving the entire colon.

The histological examination shows a modest and diffuse ischemic injury involving the entire colon. A mild hyalinization of lamina propria, a loss of superficial epithelium and ghost of crypts, with dilation and congestion of mucosal capillaries, have been seen (see Figure 2).

The histological examination, not compatible with a vascular injury, and the absence of signs of infection have allowed a diagnosis of drug-induced ischemic colitis secondary to drugs use.

Symptoms regressed after some day reducing steroid therapy (0.2 mg/Kg/die of Prednisone) and the patient did not require any blood transfusion.

## 3. Conclusion

Ischemic colitis is the most common form of ischemic injury of the gastrointestinal tract [2]. It is was first reported by Boley et al. in 1963 as a reversible ischemic disorder that occurs in the small vasculature of the colon without the obliteration of major mesenteric trunks [3]. 

There are many risk factors for ischemic colitis, ranging from atherosclerotic and valvular heart disease to postsurgical conditions and hypercoagulable states. Also pharmacological agents can induce alterations of colonic blood flow and induce ischemic colitis.

With immunosuppressive treatment, there are reported few cases of ischemic colitis, induced by immunomodulators like interleukin-2 (IL-2) or interferon (IFN) [4], sodium aurothiomalate [5], and steroid therapy also associated with azathioprine.

There are only two cases described in the literature with steroid therapy: the first by Yamanishi et al. in 1996 reported a case of ischemic colitis associated with steroid pulse therapy in an adult patient with progressive systemic sclerosis [6]. 

The second case by Yanagisawa et al. in 2008 reported a case of a young man with steroid-dependent nephrotic syndrome who developed ischemic colitis after methylprednisolone and mizoribine pulse therapy [7].

Only Gomella et al. in 1986 described the effect of immunosuppressive agents (methylprednisolone and/or azathioprine) on the development of ischemic colitis in rats [8].

In conclusion, we report the first case of an ischemic pancolitis resembling IBD at colonoscopy. Histology was necessary in establishing the diagnosis.

Steroid treatment could induce ischemic colitis by inducing a hypercoagulative state, but the exact mechanism remains unclear. To our knowledge, azathioprine has never been associated with ischemic colitis development.

So patients likely to be prescribed these medical treatments should be carefully monitored for any sign of ischemic colitis.

## References

[1] Brandt LJ, Feldman M, Friedman LS, Brandt LJ (2006). Intesctinal ischemia. *Sleisenger and Fordtran's Gastrointestinal and Liver Disease*.

[2] Feuerstadt P, Brandt LJ (2010). Colon ischemia: recent insights and advances. *Current Gastroenterology Reports*.

[3] Boley SJ, Schawartz S, Lash J, Sternill V (1963). Reversible vascular occlusion of the colon.. *Surgery, Gynecology & Obstetrics*.

[4] Sparano JA, Dutcher JP, Kaleya R (1991). Colonic ischemia complicating immunotherapy with interleukin-2 and interferon-alpha. *Cancer*.

[5] Wright A, Benfield GFA, Felix-Davies D (1984). Ischaemic colitis and immune complexes during gold therapy for rheumatoid arthritis. *Annals of the Rheumatic Diseases*.

[6] Yamanishi Y, Yamana S, Ishioka S, Yamakido M (1996). Development of ischemic colitis and scleroderma renal crisis following methylprednisolone pulse therapy for progressive systemic sclerosis. *Internal Medicine*.

[7] Yanagisawa A, Namai Y, Sekine T, Igarashi T (2008). Ischemic colitis as a complication in a patient with steroid-dependent nephrotic syndrome. *Pediatric Nephrology*.

[8] Gomella LG, Gehrken A, Hagihara PF, Flanigan RC (1986). Ischemic colitis and immunosuppression. An experimental model. *Diseases of the Colon and Rectum*.

